# Intranasal insulin and postoperative delirium in adult surgical patients: a meta-analysis and systematic review of randomized controlled trials

**DOI:** 10.3389/fmed.2025.1670982

**Published:** 2025-11-12

**Authors:** Li-cai Zhang, Jian-li Song, Li-quan Qiu, Qiang Li, Xuan Yu, Bin Lu, Guan-yu Chen

**Affiliations:** Departments of Anesthesiology, Zigong Fourth People's Hospital, Zigong, Sichuan, China

**Keywords:** meta-analysis, postoperative delirium, systematic review, intranasal insulin, hypoglycemia

## Abstract

**Introduction:**

The efficacy and safety of intranasal insulin (INI) for preventing postoperative delirium (POD) remain uncertain.

**Methods:**

We searched PubMed, Web of Science, Cochrane Library, Embase, and registers from inception to July 1, 2025, for randomized controlled trials (RCTs) enrolling adult surgical patients that compared INI with control (saline) investigating the efficacy of INI for POD prevention. The risk of bias was assessed using the revised Cochrane Risk of Bias tool (RoB 2), and the certainty of evidence was evaluated with the GRADE framework. Primary and secondary outcomes were POD incidence and a comprehensive set of secondary measures (including cognitive scores, hypoglycemia rates, pain scores, and inflammatory markers), respectively.

**Results:**

A meta-analysis of 7 randomized trials (*n* = 765) showed that INI significantly reduced the incidence of POD within 3 days postoperatively (RR = 0.35; 95% CI: 0.26–0.46; *P* < 0.001; *I*^2^ = 0%) and improved cognitive recovery (MMSE mean difference = 0.99; 95% CI: 0.52–1.47; *P* < 0.001; *I*^2^ = 1.7%). INI also reduced early postoperative interleukin-6 (IL-6) levels without affecting the incidence of hypoglycemia or pain scores.

**Conclusion:**

INI may protect perioperative cognitive function, reduce POD incidence within 3 days postoperatively, and alleviate postoperative inflammation without increasing hypoglycemia risk. However, larger-scale, randomized, multicenter trials are needed to confirm clinical efficacy and establish optimal protocols.

**Clinical trial registration:**

The protocol for this meta-analysis is available in PROSPERO (CRD42024614995).

## Introduction

Postoperative delirium (POD) is a prevalent complication observed in elderly patients following surgical procedures, characterized primarily by cognitive disturbances, disorientation, inattention, reversal of day-night cycles, and alterations in consciousness (1). The incidence of POD in this demographic ranges from 15 to 62%, with rates escalating to as high as 80% among elderly individuals admitted to intensive care units (2). In elderly patients undergoing complex heart surgery, POD can occur in up to one-third of cases (3). The occurrence of POD is associated with increased complications, mortality rates, and prolonged hospital stays (4, 5). However, the pathophysiological mechanism of POD remains unclear, and prevention is currently the most effective strategy to reduce its occurrence. Therefore, it is crucial to explore an effective, cost-efficient and feasible intervention to prevent the development of postoperative delirium.

Intranasal insulin administration has emerged as a significant approach for addressing cognitive impairments linked to abnormal brain energy metabolism, garnering considerable attention in recent years (6–8). A substantial body of experimental and clinical evidence supports the effectiveness of intranasal insulin in treating chronic neurodegenerative diseases such as Alzheimer's disease, Parkinson's disease, mild cognitive impairment, and neuropathy (9–11). The brain is particularly responsive to insulin, with insulin receptors being extensively distributed throughout the organ, particularly concentrated in the cortical marginal structures (12). The intranasal delivery of insulin allows for its transport to the brain via olfactory and trigeminal pathways, resulting in a rapid increase in cerebral insulin levels and subsequent stimulation of insulin signaling pathways in neurons (13, 14). The neuroprotective mechanisms of insulin are multifaceted, involving critical pathways such as PI3K/Akt and MAPK/ERK, which are vital for cellular survival and synaptic integrity (15). Additionally, insulin enhances glucose uptake and metabolism in the brain, regulating synaptic plasticity and neurotransmitter release, which are essential for cognitive processes related to learning and memory (15). Based on this mechanistic rationale and evidence from chronic neurodegenerative diseases, researchers have begun investigating intranasal insulin for the prevention of acute postoperative delirium. Consequently, several randomized controlled trials have been conducted; however, their findings are potentially heterogeneous or individually underpowered to definitively establish the optimal dosage, timing of administration, efficacy, and safety (16–21).

Therefore, it is essential to conduct a meta-analysis and systematic review to assess the efficacy and safety of INI vs. normal saline control for preventing POD in adult surgical patients, thereby providing evidence for clinical POD prevention.

## Methods

This study was performed according to the Preferred Reporting Items for Systematic Reviews and Meta-Analyses (PRISMA) statement (23). The protocol for this meta-analysis is available in PROSPERO (CRD42024614995) (24).

### Search for trials

Two independent authors (ZLC and SJL) systematically searched PubMed, Web of Science, the Cochrane Library, and Embase from their inception to July 1, 2025, using keywords including “delirium,” “postoperative delirium,” “insulin,” and “intranasal insulin” to identify studies meeting the inclusion criteria. Additionally, we searched ClinicalTrials.gov, the World Health Organization approved trial registry, and the OpenGrey database to ensure the comprehensive inclusion of ongoing or unpublished studies. Furthermore, to prevent the omission of relevant studies, the reference lists of included studies and the published systematic reviews were screened. Any discrepancies were resolved through discussing with another author (CGY). The language was limited to English. The search strategy is listed in Supplementary File 1.

### Selection criteria and inclusion criteria

The inclusion criteria were as follows: (a) Population: adults patients scheduled for surgery; (b) interventions: INI administered via intranasal instillation/spray; (c) comparison: Intranasal normal saline, with the same administration route and frequency as the intervention group; (d) outcome: data on the incidence of POD; and (e) study design: RCT. Included studies must have a follow-up period of at least 3 days postoperatively to fully record POD incidence within 3 days after surgery.

The exclusion criteria were as follows: (1) observational studies, reviews, case reports, conference papers, studies with incomplete or unavailable data, and animal studies; (2) Studies that failed to measure or report primary outcome (POD incidence) were excluded; (4) incomplete outcome data or inaccessible protocols precluding quantitative synthesis; (5) publications in languages other than English.

### Data extraction

Data extraction was performed independently by two authors (ZLC and SJL), with any disagreements resolved by a third researcher (CGY). The extracted data encompassed basic study characteristics (first author, year, country, design, sample size), POD assessment details (scale and timepoint within 3 days postoperatively), and predefined outcomes. The primary outcome was POD incidence. Secondary outcomes included: cognitive function (MMSE scores at baseline and within 3 days postoperatively); pain scores (VAS/NRS on days 1, 2, and 3); glucose metabolism (fasting glucose preoperatively and on postoperative day 1); and inflammatory markers (IL-6 and TNF-α on postoperative day 1). Surgery type was categorized as cardiac or non-cardiac, with specific procedures recorded.

### Quality of evidence and sensitivity analyses

Two authors (ZLC and SJL) independently evaluated the risk of bias using the revised Cochrane risk of bias tool for RCTs (RoB 2) (25). This tool encompasses the assessment of five domains, namely the randomization process, deviations from intended interventions, missing outcome data, measurement of the outcome, and selection of the reported result. Each domain was assigned a classification of low risk, some concerns, or high risk of bias. The certainty of the evidence was assessed using the Grading of Recommendations Assessment, Development, and Evaluation (GRADE) framework (26). Final findings were generated using GRADEpro software (gradepro.org) (27). Disagreements were resolved by a third researcher (CGY), who made the final decision. Sensitivity analyses were used to establish the reliability and quality of the results by iteratively omitting single studies.

### Statistical analysis

All statistical analysis was performed using STATA software version 14.0 (StataCorp, College Station, TX). For the continuous variables, the mean difference (MD) and the standardized mean difference (SMD) with 95% confidence intervals (CI) were calculated. The SMD was used if different scales or measurement methods were employed between studies to measure the same outcomes, while MD was used when the outcomes of all studies included in the meta-analysis adopted the same measurement method. For dichotomous variables, the risk ratio (RR) with 95% CI was pooled. The *I*^2^ statistic was used to analyze heterogeneity, and pooled analyses of outcomes were performed with a random-effects model in the presence of significant heterogeneity (*I*^2^ ≥ 50%), with fixed-effects models otherwise being utilized.

To account for statistical or clinical heterogeneities, subgroup analysis was performed according to delirium scale, anesthesia method, type of surgery, dosage of insulin and age. To evaluate the robustness of the pooled results, we performed the following sensitivity analyses. First, a leave-one-out analysis was conducted by iteratively excluding each individual study and re-estimating the pooled risk ratio (RR) with 95% confidence intervals to assess whether any single study disproportionately influenced the overall effect size. Second, we carried out subgroup-based sensitivity analyses stratified by key covariates, including postoperative care protocols, research team, anesthesia techniques, and diabetic status. These analyses examined whether effect estimates remained consistent across subgroups and identified potential effect modifiers. When the number of included studies was 10, the publication bias was assessed by funnel plots and Egger's test.

## Results

### Studies retrieved and their characteristics

**Figure 1:**
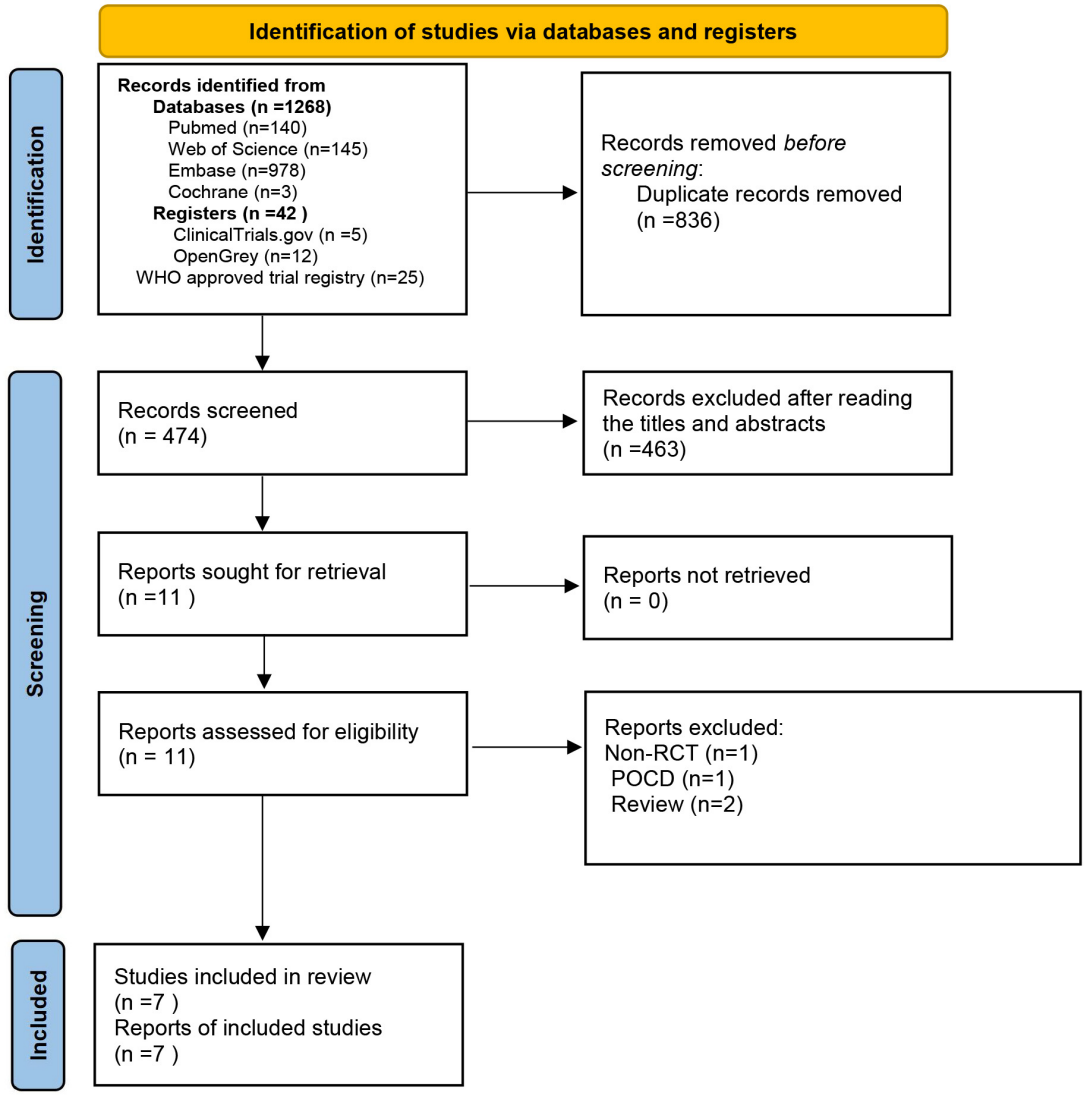
Flow chart for study selection.

The database search yielded 1,310 records that were deemed potentially eligible for inclusion. After eliminating duplicates, we reviewed 474 records by examining their titles and abstracts. Out of these, 463 records were discarded due to being unrelated to the topic, while 11 records satisfied the inclusion criteria. A further 4 publications were excluded for the following reasons: one was identified as a non-randomized controlled study, two were categorized as review articles, and one was classified as pertaining to postoperative cognitive dysfunction (POCD). Eventually, seven studies were included in the meta-analysis (16–22) (Figure 1).

The characteristics of the included studies are summarized in Table 1. All trials incorporated participants from China. The sample sizes in the meta-analysis ranged from 71 to 195 subjects, resulting in a total of 765 participants. Of these, 418 individuals were allocated to the intranasal insulin group, whereas 347 individuals were assigned to the normal saline group. In terms of postoperative delirium (POD) incidence, 182 (23.8%) of the 765 participants were diagnosed with POD, and 583 (76.2%) had no POD. Specifically, the incidence of POD was 13.1% in the INI group, compared with 36.6% in the control group.

**Table 1:** Characteristics of the included studies.

**Author (year)**	**Country**	**Age**	**Type of surgery**	**Study design**	**Type of anesthesia**	**Number of patients**	**Intervention characteristics**		**Delirium Assessment Scale**	**POD evaluation time**	**Outcomes**
							**Insulin pretreatment group**	**Control group**			
Huang 2021	China	Age ≥ 60 years	Laparoscopic radical gastrointestinal surgery	RCT	GA	80	Starting 2 days before surgery, administer 0.5 mL (20 U) of INS intranasally twice a day (*n* = 40)	Saline control (*n* = 40)	CAM-ICU	Five days postoperatively	POD, TNF-α, IL-1β, IL-6, NRS score, adverse events
Huang 2023	China	Age ≥ 65 years	Radical esophageal cancer surgery	RCT	GA	90	Starting 2 days before surgery, administer 0.5 mL (20 U, n1 = 30) or 0.75 ml (30 U, n2 = 30) of INS intranasally twice a day	Saline control (*n* = 30)	CAM-ICU	Three days postoperatively	POD, τ protein and Aβ protein, NRS, adverse events
Huang 2024	China	18–65 years	Valve replacement surgery with CPB	RCT	GA	71	Starting 2 days before surgery, administer 0.5 ml (20 U) of INS intranasally twice a day (*n* = 36)	Saline control (*n* = 35)	CAM-ICU	Three days postoperatively	POD, serum cortisol, sleep quality, NRS, adverse events
Li 2025	China	Age ≥ 65 years	elective unilateral hip arthroplasty or closed reduction and intramedullary nailing	RCT	Spinal anesthesia	130	INS intranasally at 19:00 on the day before surgery, 50 min before anesthesia on the day of surgery and at 19:00 on the day of surgery, INS 20U (n1 = 45) or INS 40U (n2 = 42)	Saline control (n = 43)	CAM-ICU	Three days postoperatively	POD, VAS, Glucose and lactate levels in the CSF, adverse events
Sun 2024	China	Age ≥ 65 years	Elective orthopedic surgery or pancreatic surgery	RCT	GA	128	5 min before anesthesia induction and on the first through third days after surgery, administer 40 IU of INS intranasally once a day (*n* = 64)	Saline control (*n* = 64)	3D-CAM	Three days postoperatively	POD, perioperative cognitive function, IL-6, TNF-α, S100β, insulin resistance
Yang Mi 2025	China	65–95 years	Elective joint replacement surgery	RCT	Combined epidural anesthesia	195	Intranasal sprays in the morning and night with 40 IU of INS (1 mL) twice a day from the 3rd day before surgery to the 5th day after surgery (*n* = 96)	Saline control (*n* = 99)	CAM and DRS-98	Five days postoperatively	POD, BNDF, insulin and glucose concentrations in plasma and CSF
Yang Ming 2025	China	45–65 years	Elective cardiac surgery with CPB	RCT	GA	71	INS intranasally at a dose of 0.5 mL containing 20 IU 1 h before surgery in the operating room and at 8:00 a.m. on postoperative days 1 and 2 (*n* = 35)	Saline control (*n* = 36)	CAM-ICU, CAM, 4-AT	Five days postoperatively	POD, the length of ICU and hospital stay, in-hospital mortality, and incidence of POCD at 1 month, glucose and lactate levels, VAS, sleep quality

RCT, Randomized controlled trial; GA, General Anesthesia; CPB, Cardiopulmonary Bypass; INS, Insulin; CAM-ICU, Confusion Assessment Method for the Intensive Care Unit; 3D-CAM, 3-Minute Diagnostic Interview for Confusion Assessment Method; DRS-98, Delirium Rating Scale-98; POD, Postoperative Delirium; TNF-α, Tumor Necrosis Factor-α; IL-1β, Interleukin-1β; IL-6, Interleukin-6; NRS, Numerical Rating Scale; VAS, Visual Analog Scale; CSF, Cerebrospinal Fluid; BDNF, Brain-Derived Neurotrophic Factor; S100β, S100 Calcium-Binding Protein β; POCD, Postoperative Cognitive Dysfunction; ICU, Intensive Care Unit; 4-AT, 4 A's Test.

In the assessment of delirium, five studies adopted the Confusion Assessment Method for the Intensive Care Unit (CAM-ICU) scale, one used the Confusion Assessment Method (CAM) scale, and one employed the 3-Minute Diagnostic Interview for Confusion Assessment Method (3D-CAM) scale. Regarding the surgical procedures investigated, two studies focused on cardiac surgeries, whereas the other five centered on non-cardiac surgeries.

For outcome measures, all trials evaluated the incidence of POD. Five trials assessed postoperative pain scores, and two analyzed postoperative serum levels of interleukin-6 (IL-6) and tumor necrosis factor-alpha (TNF-α). Two studies reported changes in Mini-Mental State Examination (MMSE) scores before and after surgery. Furthermore, five studies documented perioperative blood glucose levels, and five trials recorded the incidence of hypoglycemia.

### Bias risk assessment

**Figure 2:**
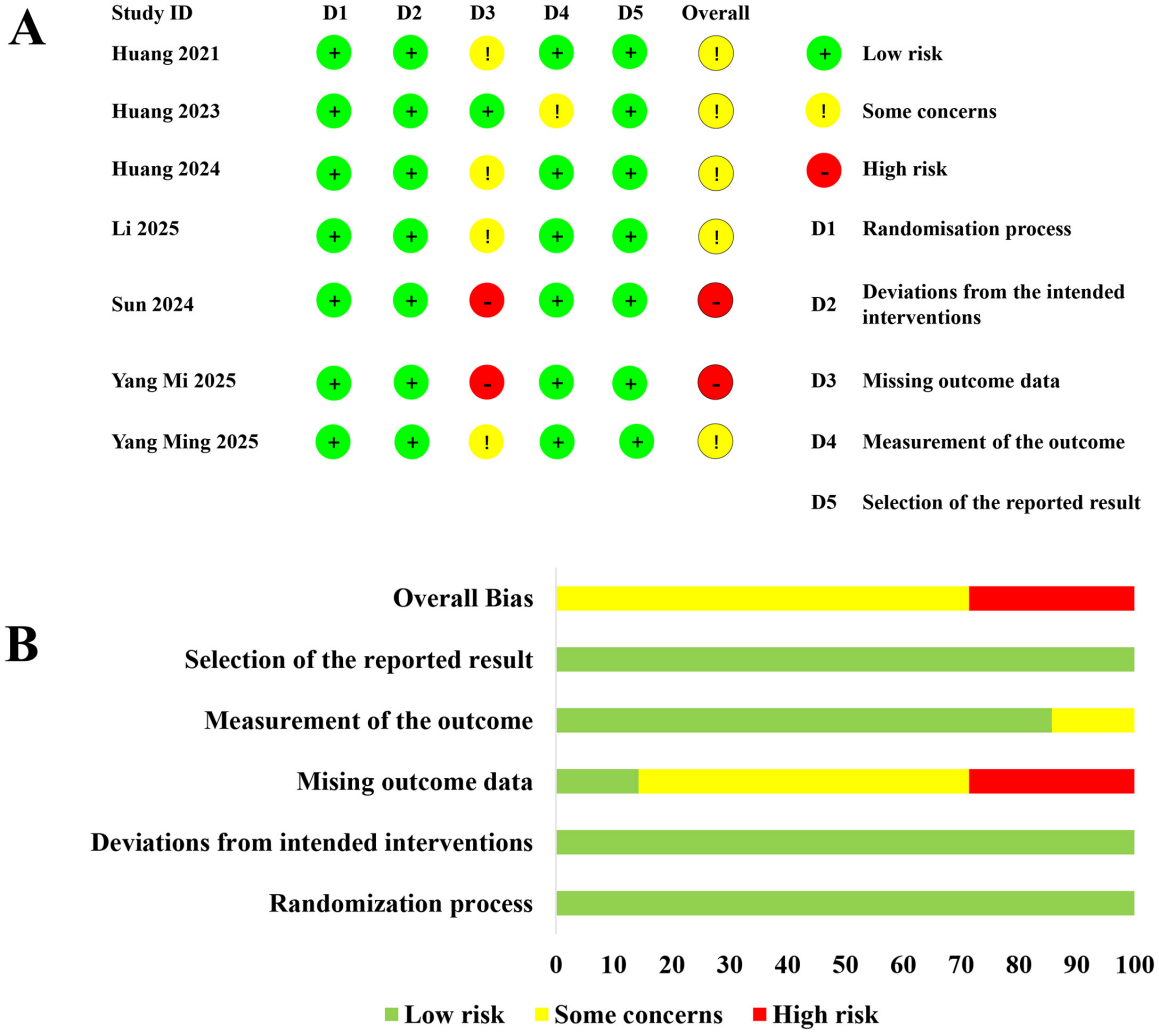
Summary of the risk of bias assessment. **(A, B)** showing risk assessment across various studies. **(A)** lists seven studies with domains D1 to D5, using green for low risk, yellow for some concerns, and red for high risk. **(B)** displays aggregated risk percentages for each domain, with varying lengths of colored bars depicting the level of risk.

The overall risk-of-bias assessment showed that 5 studies had some concerns, while two studies had high risk (Figure 2). The GRADE evaluation indicated that the evidence was of moderate certainty for outcomes including POD incidence, MMSE scores, preoperative glucose levels, postoperative glucose levels, and pain scores on postoperative day 2. Conversely, GRADE revealed low-certainty evidence for pain scores on postoperative day 1 and 3, primarily due to high heterogeneity and bias risk (Supplementary Table 1).

### Effect of intranasal insulin on postoperative delirium

**Figure 3:**
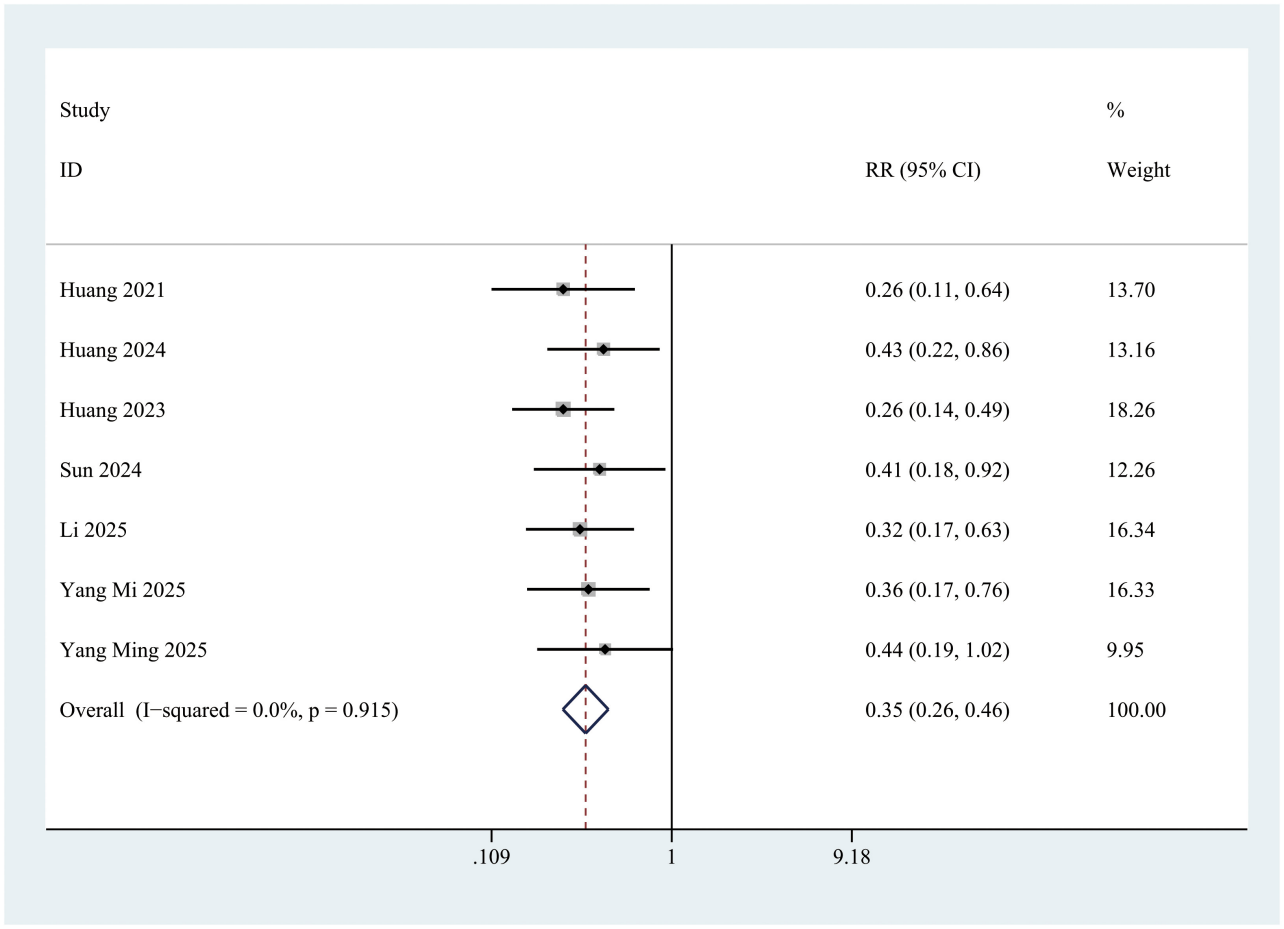
Forest plot of incidence of postoperative delirium within 3 days of surgery in control group and Insulin group. INI group: insulin group. Overall effect Z = 8.21, *P* < 0.001.

**Figure 4:**
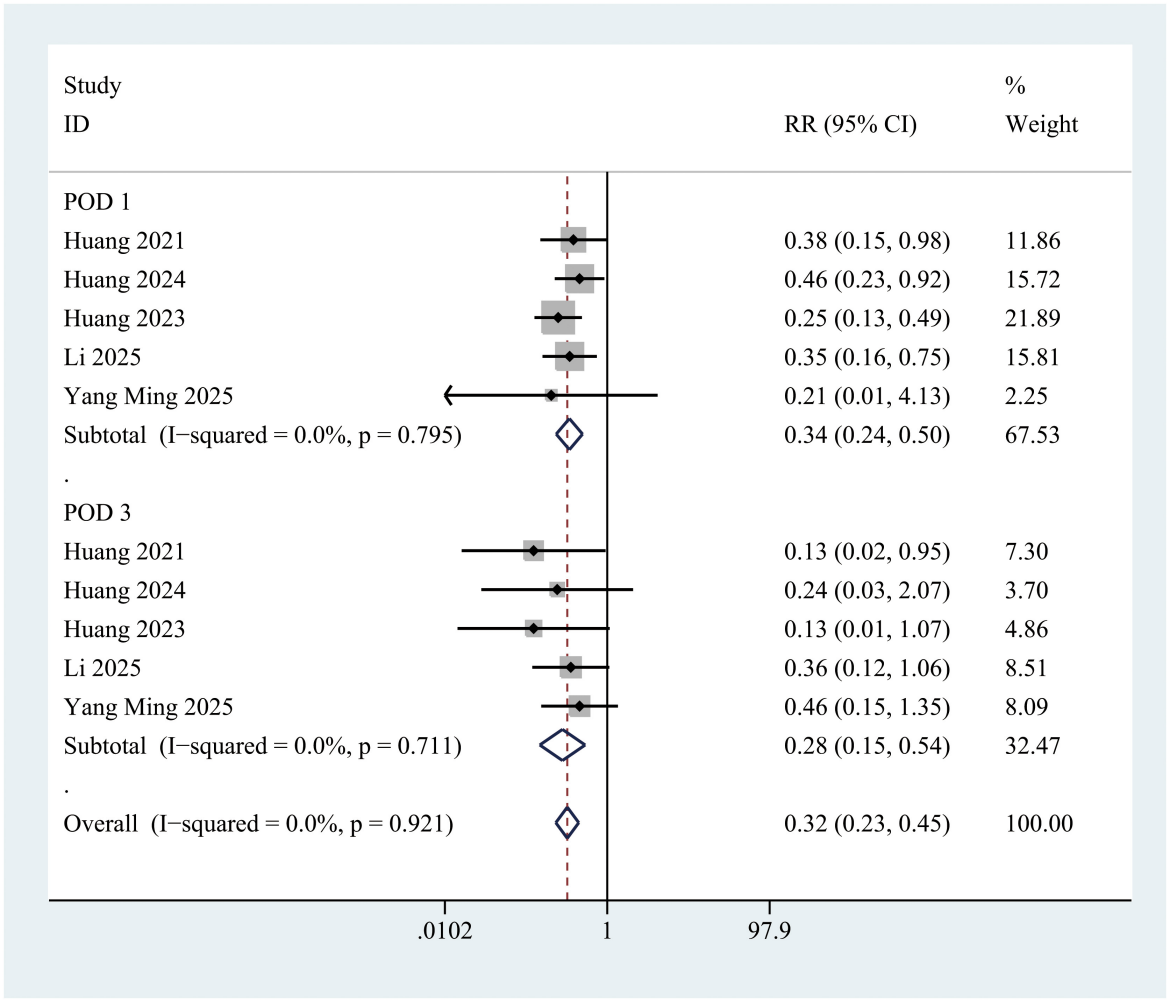
Forest plot of incidence of postoperative delirium in control group and Insulin group at postoperative day 1 and postoperative day 3. POD on postoperative day 1: Z = 6.35, *P* < 0.001; POD on postoperative day 3: Z = 3.98, *P* < 0.001.

The incidence of postoperative delirium (POD) within the first 3 days was significantly lower in the INI group compared to controls (RR = 0.35; 95% CI 0.26–0.46; *P* < 0.001; *I* = 0%) (Figure 3). This significant reduction persisted at postoperative day 1 (RR = 0.34; 95% CI 0.24–0.50; *P* < 0.001; *I*^2^ = 0%) and day 3 (RR = 0.28; 95% CI 0.15–0.54; *P* < 0.001; *I*^2^ = 0%) (Figure 4).

### Effect of intranasal insulin treatment on mini-mental state examination scores

**Figure 5:**
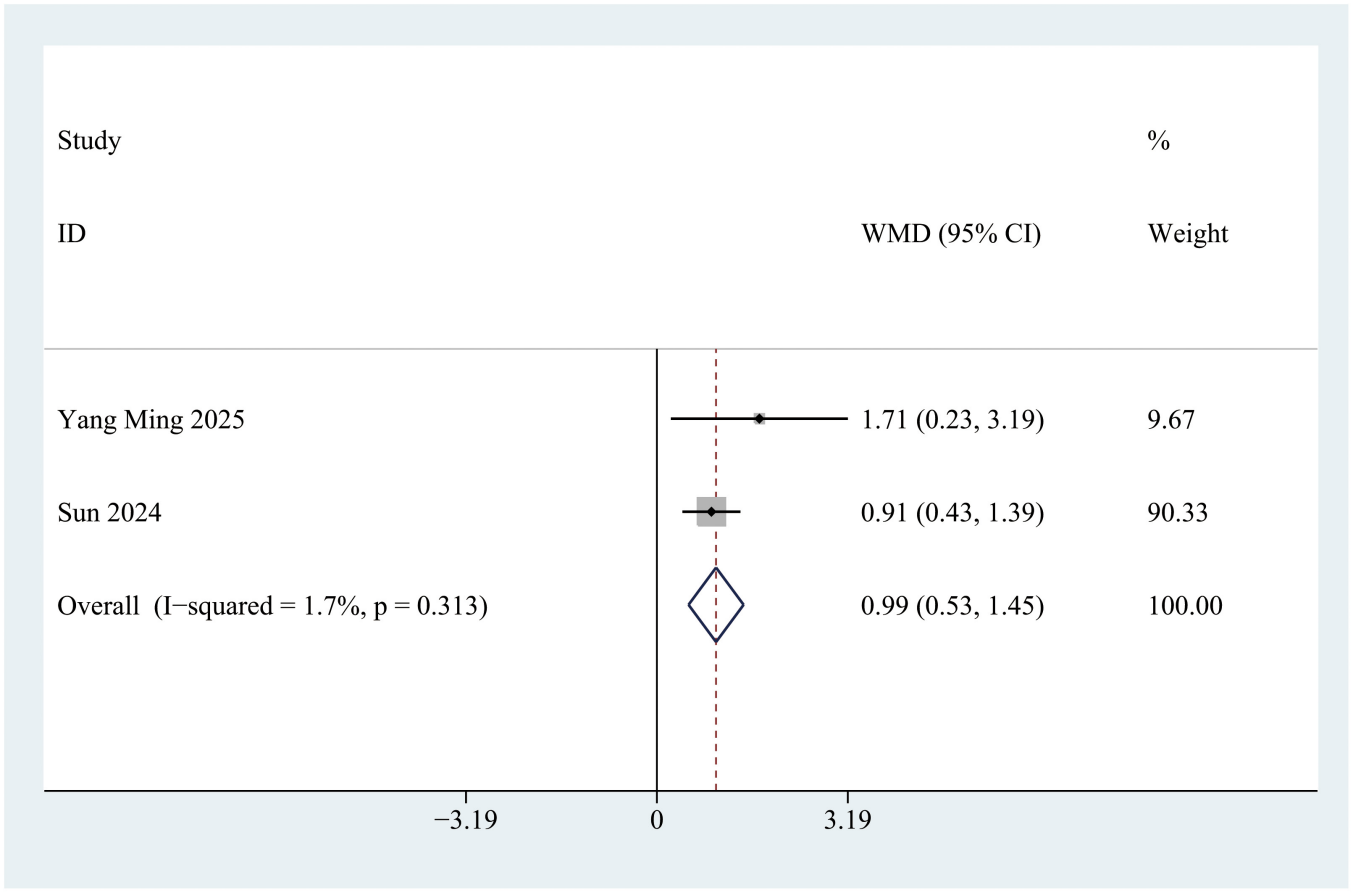
Forest plot of Mini-Mental State Examination (MMSE) scores in control group and Insulin group. Z = 4.15, *P* < 0.001.

Two studies documented changes in Mini-Mental State Examination (MMSE) scores during the perioperative period. A statistically significant difference in perioperative MMSE score changes was observed between the INI group and the control group [Mean Difference (MD) = 0.99; 95% Confidence Interval (CI): 0.52 to 1.47; *P* < 0.001; *I*^2^ = 1.7%] (Figure 5). This improvement is considered clinically meaningful, as previous studies have shown that a postoperative increase in MMSE score of ≥0.5 points is associated with a significant reduction in the risk of long-term cognitive dysfunction.

### Effect of intranasal insulin treatment on postoperative pain

The postoperative pain intensity was similar between the two groups on postoperative day 1 (SMD = −0.36; 95% CI −0.77 to 0.05; *P* = 0.084; *I*^2^ = 81%), postoperative day 2 (SMD = 0; 95% CI −0.19 to 0.19; *P* = 0.987; I^2^ = 0%) and postoperative day 3 (SMD = 0.42; 95% CI −0.43 to 1.27; *P* = 0.334; *I*^2^ = 95.2%) (Supplementary Figure 1).

### Comparison of blood glucose levels between control group and INI group before and after surgery

No significant glucose differences existed preoperatively (WMD = 0; 95% CI −0.16 to 0.16; *P* = 0.995; *I*^2^ = 17.6%) or postoperatively (WMD = −0.02; 95% CI −0.27 to 0.24; *P* = 0.895; *I*^2^ = 16.5%) (Supplementary Figure 2). Five trials documented postoperative hypoglycemia, though no patients developed symptomatic hypoglycemia.

### Effect of intranasal insulin treatment on perioperative biomarkers for inflammation

Compared with controls, INI significantly reduced IL-6 levels on postoperative day 1 (WMD = −3.57; 95% CI −4.68 to −2.46; *P* < 0.001; *I*^2^ = 0%). TNF-α levels showed no significant intergroup difference (Supplementary Figure 3).

### Subgroup analyses

Subgroup analysis was performed according to delirium scale, anesthesia method, type of surgery, dosage of insulin and age (Supplementary Figures 4–8). Subgroup analysis demonstrated that INI consistently reduced the incidence of POD across all evaluated subgroups: regardless of whether delirium was assessed using the CAM-ICU (RR = 0.33; 95% CI 0.24–0.46; *P* < 0.001; *I*^2^ = 0%) or other scales (RR = 0.38; 95% CI 0.22–0.66; *P* = 0.001; *I*^2^ = 0%), in patients undergoing general anesthesia (RR = 0.35; 95% CI 0.25–0.49; *P* < 0.001; *I*^2^ = 0%) or non-general anesthesia (RR = 0.34; 95% CI 0.21–0.56; *P* < 0.001; *I*^2^ = 0%), in those undergoing cardiac (RR = 0.44; 95% CI 0.26–0.74; *P* = 0.002; *I*^2^ = 0%) or non-cardiac surgery (RR = 0.32; 95% CI 0.23–0.44; *P* < 0.001; *I*^2^ = 0%), in individuals aged over 65 (RR = 0.32; 95% CI 0.23–0.44; *P* < 0.001; *I*^2^ = 0%) or under 65 (RR = 0.44; 95% CI 0.26–0.74; *P* = 0.002; *I*^2^ = 0%), and with insulin dosages of either 20 IU (RR = 0.39; 95% CI 0.28–0.55; *P* < 0.001; *I*^2^ = 0%) or 40 IU (RR = 0.38; 95% CI 0.24–0.60; *P* < 0.001; *I*^2^ = 0%), the intranasal insulin group showed a lower incidence of POD compared to the control group.

### Sensitivity analysis and publication bias

The sensitivity analyses revealed that no single study unduly influenced the overall risk ratio (RR) and its 95% confidence intervals (Supplementary Figure 9). Furthermore, analyses stratified by postoperative care, research teams, anesthesia techniques, and diabetic status demonstrated consistent intervention effects with no significant differences across subgroups, confirming the robustness of the findings (Supplementary Table 2). Funnel plot was unsuitable for assessment of publication bias because the number of included studies was seven.

## Discussion

As the first meta-analysis investigating the effect of intranasal insulin on postoperative delirium (POD) in adult surgical patients, this study demonstrates that intranasal insulin significantly reduces POD incidence, promotes cognitive recovery, and does not increase hypoglycemia risk. Subgroup analyses further revealed that this beneficial effect was consistent across various subgroups—regardless of the delirium assessment tool (CAM-ICU or others), anesthesia type (general or non-general), surgery type (cardiac or non-cardiac), patient age (< 65 or ≥65 years), or insulin dosage (20 IU or 40 IU). These results suggest that intranasal insulin represents a novel and promising intervention for POD prevention in adult surgical patients.

However, postoperative pain scores demonstrated substantial heterogeneity on days and day 3. These results should be interpreted with caution, avoiding overinterpreting the effect of intranasal insulin on pain. This heterogeneity primarily stems from three interconnected factors. First, differences in surgical types led to varying pain baselines: gastrointestinal laparoscopic procedures were associated with milder pain, whereas cardiac valve replacement surgeries presented more significant pain levels. Additionally, hip arthroplasty cases showed pain rebound by day 3 due to early ambulation—these inherent differences contributed to considerable score dispersion. Second, diversified pain management protocols amplified efficacy variation: regional nerve blocks provided superior analgesia compared to intravenous opioids alone, and the development of opioid tolerance further widened these gaps by day 3. Third, potential discrepancies in pain assessment tools introduced additional variability: three studies utilized the Numerical Rating Scale (NRS, 0–10 points), while two employed the Visual Analog Scale (VAS, 0–100 points). Although statistical analysis incorporated standardized mean difference (SMD) conversions to address scale differences, the two instruments differ in patient comprehension—older patients particularly demonstrate reduced accuracy in interpreting VAS gradations, potentially leading to systematically higher scores. Collectively, these factors resulted in significant heterogeneity, indicating that pain score outcomes require careful interpretation. Future studies should standardize surgical categories, analgesic protocols, and assessment criteria to reduce variability.

Previous research indicates that intranasal insulin may prevent POD through multiple pathways, including modulating sleep architecture (e.g., improving sleep efficiency, increasing total sleep time), suppressing neuroinflammation (e.g., reducing IL-6, TNF-α levels), and enhancing cerebral insulin signaling (28–30). A key mechanism involves increasing brain insulin levels and significantly reducing inflammatory mediators like IL-6, TNF-α, and IL-1β, thereby improving cognitive function and providing neuroprotection (30). This meta-analysis found that INI significantly reduced postoperative IL-6 levels but had no significant effect on TNF-α. This “differential effect” may be related to the selective regulation of insulin on inflammatory signaling pathways. As previously reported (15), insulin inhibits the activation of nuclear factor-κB (NF-κB) through the PI3K/Akt pathway, and NF-κB exhibits higher sensitivity to the transcriptional regulation of IL-6, while the regulation of TNF-α requires the synergy of other signals (such as the MAPK pathway) (30). In addition, the timing of inflammatory factor detection may also contribute to this difference—included studies only measured inflammatory factors on postoperative day 1, yet the peak of TNF-αmay occur earlier (6–12 h postoperatively), which is earlier than the peak of IL-6. Future studies should adjust the detection time points (e.g., 6, 12, 24, 48 h postoperatively) to fully capture the dynamic changes of various inflammatory factors. Second, the brain insulin concentration, a key link in the mechanism of INI, remains an unaddressed gap in the current evidence. Although this study assumes that INI reaches the brain via olfactory and trigeminal pathways (13, 14), none of the seven included studies directly measured brain insulin concentration (e.g., via cerebrospinal fluid sampling or brain imaging techniques). The cognitive improvement observed in the study only indirectly infers the brain-targeting effect of INI. Basic research has shown that intranasal administration of 20–40 IU insulin can increase brain insulin concentration in rats by 2–3 times (14), but the “dose-effect threshold” between human brain insulin concentration and POD prevention remains unknown. This gap makes it difficult to provide direct evidence for the clinical recommendation of “effective doses”—for example, although subgroup analysis showed no significant difference in efficacy between 20 and 40 IU, it cannot be determined whether this is due to the fact that both doses have reached the threshold of brain insulin concentration required to prevent POD, or if there is a “ceiling effect” in insulin efficacy.

Furthermore, nocturnal sleep is crucial for maintaining cognitive function, facilitating learning, memory consolidation, and brain homeostasis (31, 32). From the perspective of physiological function, normal sleep promotes the clearance of neurotoxic waste via the glymphatic system, while sleep deprivation or disruption directly inhibits this process—not only leading to the accumulation of inflammatory mediators but also further damaging neuronal and synaptic function, ultimately triggering or exacerbating delirium (16). Specifically in the context of POD, disrupted sleep architecture directly impairs the aforementioned cognition-related processes and induces POD; meanwhile, poor sleep quality may also exacerbate neuroinflammation, further influencing the occurrence and progression of POD. From the perspective of intervention mechanisms, intranasal insulin (INI) may regulate the hypothalamic-pituitary-adrenal (HPA) axis to reduce nocturnal cortisol release, thereby promoting slow-wave sleep—a critical phase for the glymphatic system to clear neurotoxic proteins such as Aβ and tau (31, 32). Clinical evidence further supports the close association between sleep quality and POD: For instance, observational studies in patients undergoing cardiac surgery found that postoperative insomnia and circadian rhythm disturbances were significantly associated with the risk of severe POD, while active improvement of sleep architecture effectively reduced the incidence of POD (33); studies on intranasal insulin intervention further verified this association—by stabilizing postoperative sleep architecture, INI significantly reduced the risk of POD, indirectly confirming the critical role of improved sleep quality in POD prevention (22). Unfortunately, insufficient data precluded meta-analysis of sleep-related outcomes.

When comparing INI with currently commonly used pharmacological interventions for POD prevention, including dexmedetomidine and melatonin, its profile demonstrates distinct advantages. A meta-analysis of dexmedetomidine reported a risk ratio (RR) of approximately 0.59 (95% CI: 0.45–0.76) for POD reduction, which is higher than the RR of 0.35 observed for INI in the present study, suggesting potentially superior efficacy of INI (33). However, dexmedetomidine requires intravenous administration and may increase the risk of hypotension, whereas INI is non-invasive and carries no increased risk of hypoglycemia, indicating a more favorable safety profile. As for melatonin, its RR for POD reduction is approximately 0.42 (33), but it requires nighttime oral administration, which can pose adherence challenges in elderly patients. Furthermore, melatonin may interact with hepatic enzymes (e.g., CYP1A2), potentially affecting the metabolism of other drugs. In contrast, there have been no reported drug interactions associated with INI, making it more suitable for elderly patients with multiple comorbidities who are often on complex medication regimens.

It is important to note that POD prevention should not rely solely on pharmacological interventions. European Society of Anaesthesiology and Intensive Care recommend multicomponent non-pharmacological strategies as first-line measures (33). These typically involve patient and family education, orientation support, sleep promotion (e.g., using eye masks and earplugs), early mobilization, timely extubation, and nutritional support. Meta-analyses have confirmed that such bundled approaches effectively reduce POD incidence compared with routine care, with sleep management being a central component. For instance, one nurse-led intervention that incorporated music therapy, breathing exercises, and sleep protocols significantly improved postoperative sleep quality and reduced hospital stay (33). However, the fact that it did not significantly lower POD incidence in that particular study suggests that optimizing sleep alone, within a broader intervention, may still be insufficient to fully counteract the multifactorial pathophysiology of POD in all patients. This limitation underscores the need for complementary approaches. In this context, intranasal insulin adds unique value by targeting the molecular and cellular underpinnings of POD—reducing neuroinflammation, enhancing insulin signaling, and supporting neuronal metabolism—thereby offering a mechanism to augment the efficacy of non-pharmacological strategies. A key direction for future research will be to determine whether combining intranasal insulin with multicomponent non-pharmacological interventions yields superior outcomes compared to either approach alone.

Beyond non-pharmacological interventions, guidelines also advise maintaining strict hemodynamic stability (avoiding prolonged mean arterial pressure below 65 mmHg), keeping EtCO_2_ between 35 and 45 mmHg to support cerebral perfusion and oxygenation, along with monitoring cerebral oxygen saturation via tools like near-infrared spectroscopy (NIRS) as a means to lower POD risk (33). Maintaining optimal cerebral perfusion and oxygenation through careful hemodynamic and ventilation management is essential. For example, Wang et al. demonstrated a U-shaped relationship between end-tidal carbon dioxide (EtCO_2_) and regional cerebral oxygen saturation (rSO_2_), indicating that both hypocapnia and hypercapnia can impair cerebral oxygenation (34). Since reduced rSO_2_ is associated with higher POD risk, individualized ventilation strategies may help prevent cerebral hypoxia and thus support the neuroprotective effects of intranasal insulin. However, multimodal monitoring alone may not be sufficient. The recent Bottomline-CS trial, a large randomized controlled study involving off-pump coronary artery bypass patients, found that although monitoring helped maintain tissue oxygen saturation near baseline, it did not significantly reduce the incidence of major complications, including POD (35). This underscores that monitoring must be coupled with targeted interventions—such as intranasal insulin, which acts on specific pathways like neuroinflammation and insulin resistance—to meaningfully improve outcomes. Therefore, integrating intranasal insulin into a comprehensive perioperative cognitive protection strategy holds great promise. Combining its anti-inflammatory and metabolic benefits with optimized ventilation, individualized blood pressure management, and multicomponent non-pharmacological interventions could simultaneously address multiple POD risk factors. Future studies should explore such multimodal approaches to determine whether combined strategies yield superior cognitive protection compared with isolated interventions.

This meta-analysis has several limitations. First, this meta-analysis included only seven randomized controlled trials. Although the total sample size reached a certain scale, the limited number of studies, all conducted in a single country, may restrict the generalizability and external validity of the findings to other populations or healthcare systems. There is an urgent need for larger international multicenter trials to further validate the efficacy and safety of intranasal insulin across diverse populations and clinical settings. Second, there was some clinical and methodological heterogeneity in outcome measurements across the studies. Although statistical heterogeneity was low for most primary outcomes, differences existed in delirium assessment tools, insulin dosing, timing, and treatment duration. Variations in diagnostic thresholds and applicable contexts of different scales may lead to inconsistent determination of POD incidence, thereby affecting the pooled effect estimates. Although subgroup analysis showed no significant difference in POD prevention between 20 and 40 IU doses, differences in dosing and timing may influence peak brain insulin concentration and duration, potentially causing efficacy fluctuations across studies. Nevertheless, subgroup analyses demonstrated consistent preventive effects of INI on POD across different assessment tools and insulin doses, enhancing the robustness of the results to some extent. Third, there are limitations in the quality of evidence for some outcome indicators. According to the GRADE assessment, evidence quality for primary outcomes such as POD incidence and MMSE scores was moderate, while it was low for indicators like pain scores on postoperative days 1 and 3 due to high heterogeneity and risk of bias. This suggests that these results should be interpreted cautiously in clinical translation. When applying intranasal insulin clinically, comprehensive judgment based on individual patient circumstances is necessary. For instance, for patients with high pain scores, pain management's impact on POD should not be neglected based solely on this study's results, and non-steroidal anti-inflammatory drugs or multimodal analgesia should be combined. Furthermore, due to limited evidence certainty, intranasal insulin is not recommended as a “first-line standalone strategy” for POD prevention but should serve as part of a multicomponent intervention. Additionally, existing literature suggests that INI may exert cognitive protective effects by improving postoperative sleep architecture. However, due to incomplete reporting of raw data in original studies, we were unable to quantitatively synthesize sleep-related outcomes. Future research should prioritize sleep quality as an important observational indicator to further elucidate its mediating role in INI's prevention of POD. Regarding dose-response relationship, this study also failed to draw clear conclusions. Although subgroup analyses indicated that both 20 IU and 40 IU doses effectively reduced POD risk, the lack of finer dose gradations and long-term course data prevents determination of the minimum effective dose, optimal dose range, or dose-effect trend. Future studies should systematically explore effect differences across various doses and treatment durations to provide more precise guidance for clinical dosing regimens. Finally, although the number of included studies did not meet requirements for funnel plot generation, potential publication bias should be considered. Unpublished small-sample studies with negative or null results may overestimate INI's actual effects. As related studies accumulate, formal publication bias tests should be conducted to more comprehensively assess the reliability of synthesized results.

Nevertheless, this remains the first meta-analysis investigating intranasal insulin's effect on POD in adult surgical patients. Our results suggest intranasal insulin may protect perioperative cognitive function, reduce POD incidence, mitigate postoperative inflammation, and does not increase hypoglycemia risk. These limitations highlight the need for larger, randomized, multicenter prospective trials to further validate its clinical efficacy and optimal treatment protocol. With ongoing research into dose optimization and systemic effects, central insulin delivery via the intranasal route holds promise as a novel approach for preventing and treating postoperative delirium in elderly patients.

## Conclusion

INI may protect perioperative cognitive function, reduce the incidence of POD, alleviate postoperative inflammatory responses, and does not increase the risk of hypoglycemia. However, there is still a need for larger-scale, randomized, multicenter prospective trials to further verify its clinical efficacy and optimal treatment protocols.

## Supplementary material

Supplementary Figure 1 Forest plot of postoperative pain scores within 3 days of surgery in control group and Insulin group. Postoperative day 1: Z = 1.74, *P* = 0.084; Postoperative day 2: Z = 0.02, *P* = 0.987; Postoperative day 3: Z = 1.01, *P* = 0.334.

Supplementary Figure 2 Forest plot of blood glucose levels between control group and Insulin group before and after surgery. Preoperative: Z = 0.01, *P* = 0.995; Postoperative: Z = 0.13, *P* = 0.895.

Supplementary Figure 3 Forest plot of inflammatory marker levels at postoperative day 1 in control group and Insulin group. IL-6:interleukin-6 TNF-α: tumor necrosis factor alpha. INS Group: Insulin group; Z = 6.02, *P* < 0.001.

Supplementary Figure 4 Forest plot illustrating the subgroup analysis of incidence of postoperative delirium based on age.

Supplementary Figure 5 Forest plot illustrating the subgroup analysis of incidence of postoperative delirium based on dosage of insulin.

Supplementary Figure 6 Forest plot illustrating the subgroup analysis of incidence of postoperative delirium based on delirium scale.

Supplementary Figure 7 Forest plot illustrating the subgroup analysis of incidence of postoperative delirium based on type of surgery.

Supplementary Figure 8 Forest plot illustrating the subgroup analysis of incidence of postoperative delirium based on method of anesthesia.

Supplementary Figure 9 Sensitivity analyses of the incidence of postoperative delirium within 3 days of surgery in control group and Insulin group.

Supplementary File 1 Search strategies for multiple databases.

Supplementary File 2 Supplementary Tables 1, 2.

Supplementary File 3 PRISMA checklist.
