# Supplementary figures and images for: Intranasal insulin and postoperative delirium in adult surgical patients: a meta-analysis and systematic review of randomized controlled trials
Source: Front Med (Lausanne). 2025 Nov 12;12:1670982. doi: 10.3389/fmed.2025.1670982 (PMC12647034; doi:10.3389/fmed.2025.1670982)

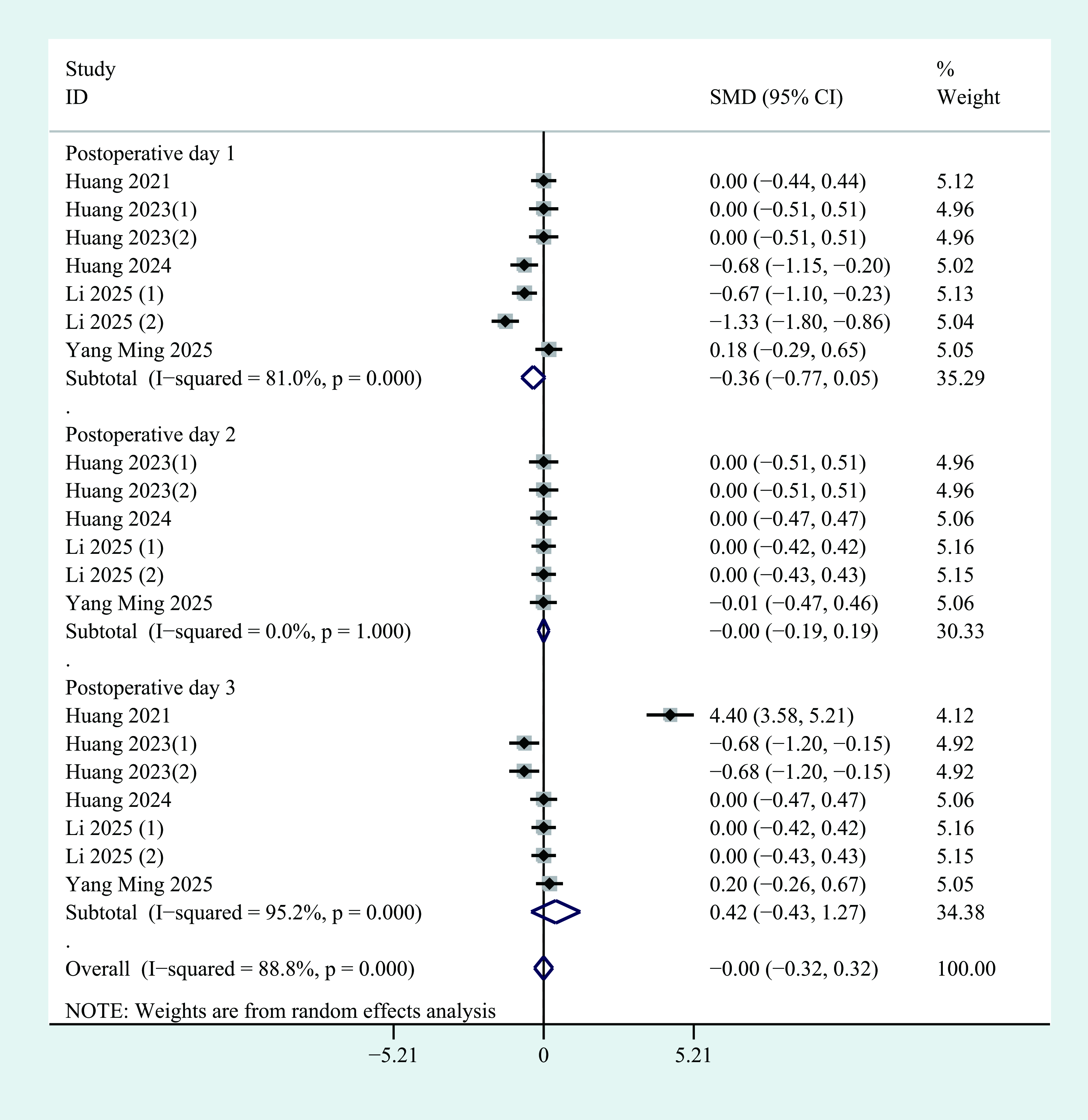

Supplement: Supplementary Figure 1 — Forest plot of postoperative pain scores within 3 days of surgery in control group and Insulin group. Postoperative day 1: Z = 1.74, P = 0.084; Postoperative day 2: Z = 0.02, P = 0.987; Postoperative day 3: Z = 1.01, P = 0.334. [file Image_1.tif]

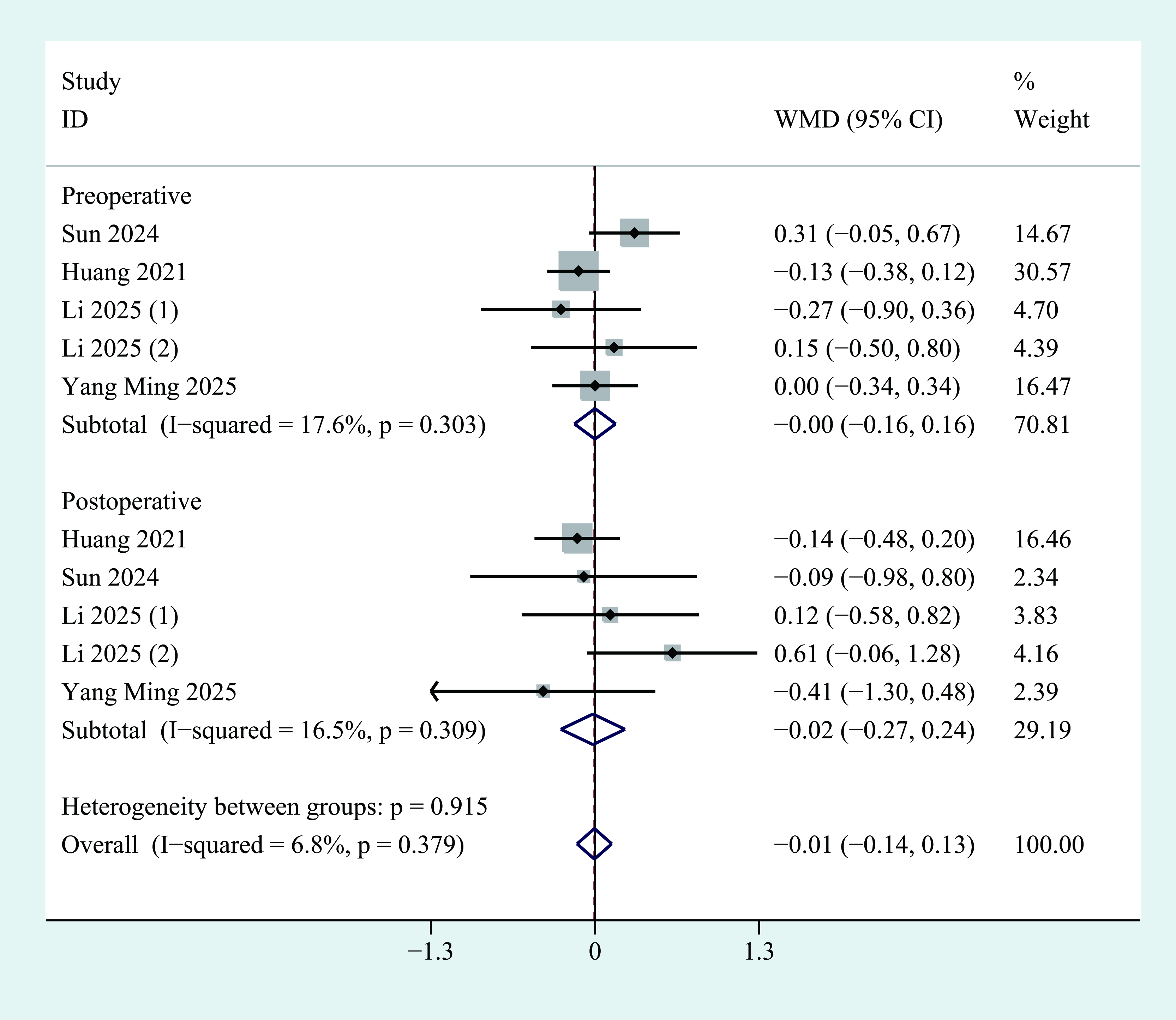

Supplement: Supplementary Figure 2 — Forest plot of blood glucose levels between control group and Insulin group before and after surgery. Preoperative: Z = 0.01, P = 0.995; Postoperative: Z = 0.13, P = 0.895. [file Image_2.tif]

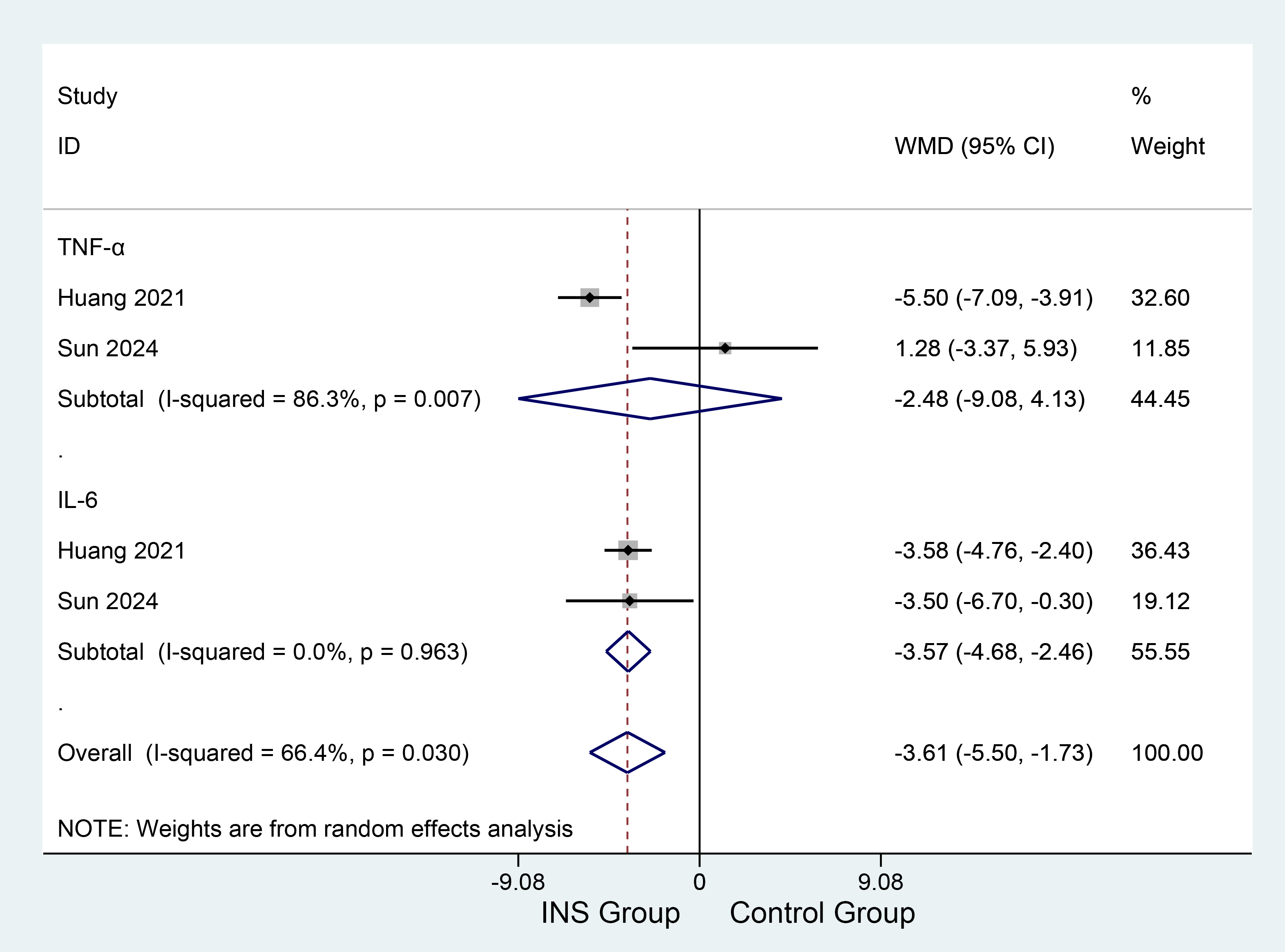

Supplement: Supplementary Figure 3 — Forest plot of inflammatory marker levels at postoperative day 1 in control group and Insulin group. IL-6:interleukin-6 TNF-α: tumor necrosis factor alpha. INS Group: Insulin group; Z = 6.02, P < 0.001. [file Image_3.tif]

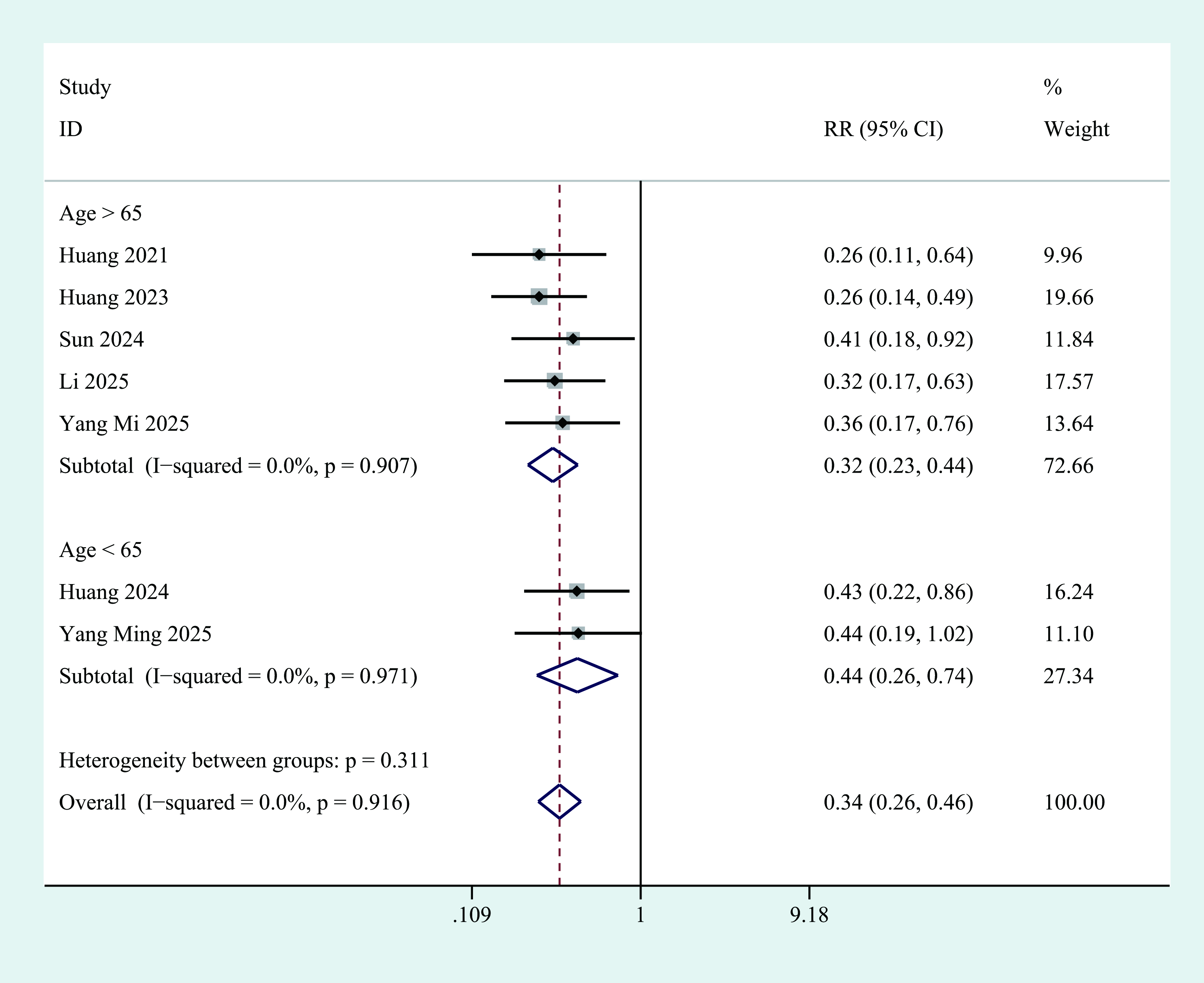

Supplement: Supplementary Figure 4 — Forest plot illustrating the subgroup analysis of incidence of postoperative delirium based on age. [file Image_4.tif]

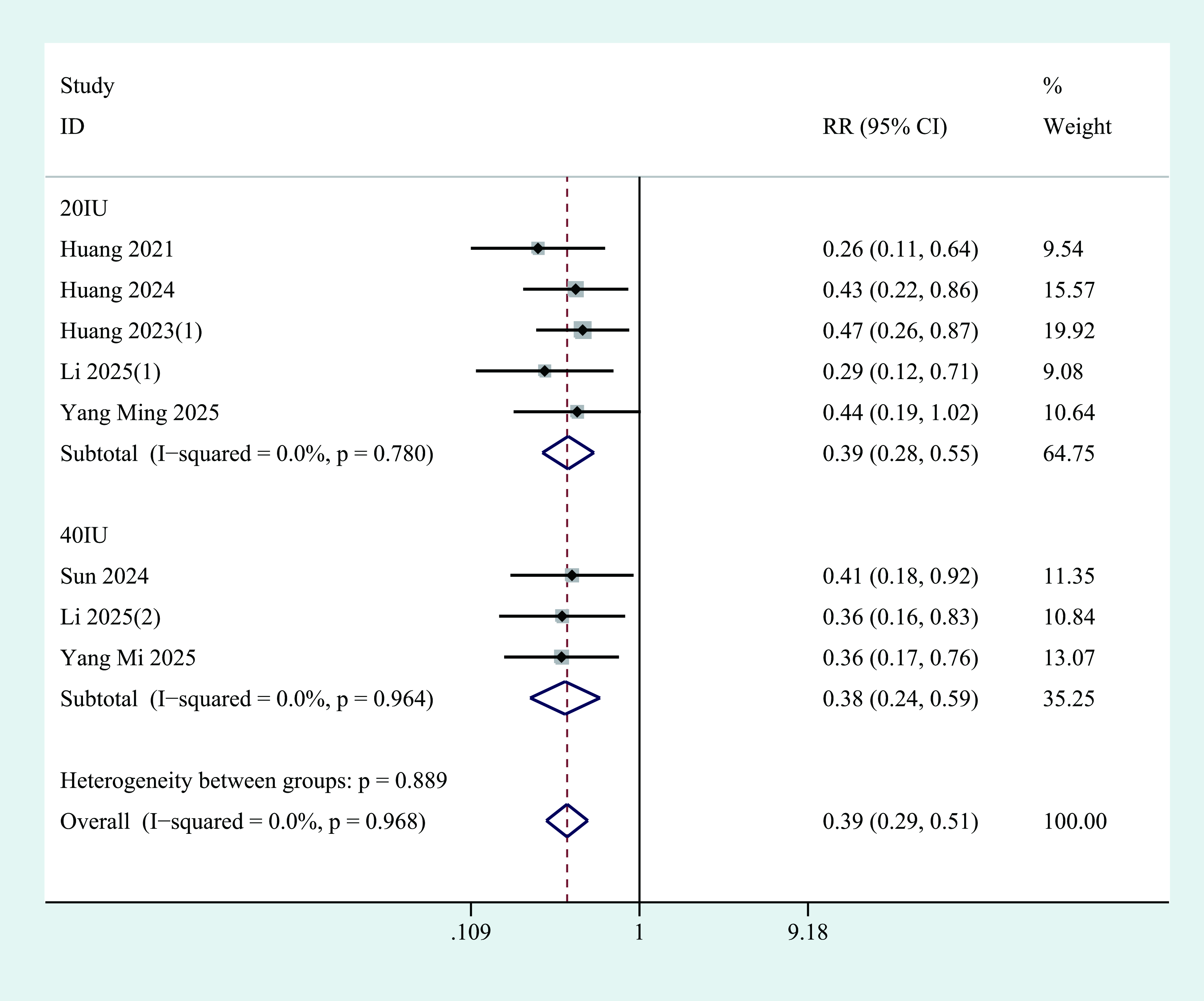

Supplement: Supplementary Figure 5 — Forest plot illustrating the subgroup analysis of incidence of postoperative delirium based on dosage of insulin. [file Image_5.tif]

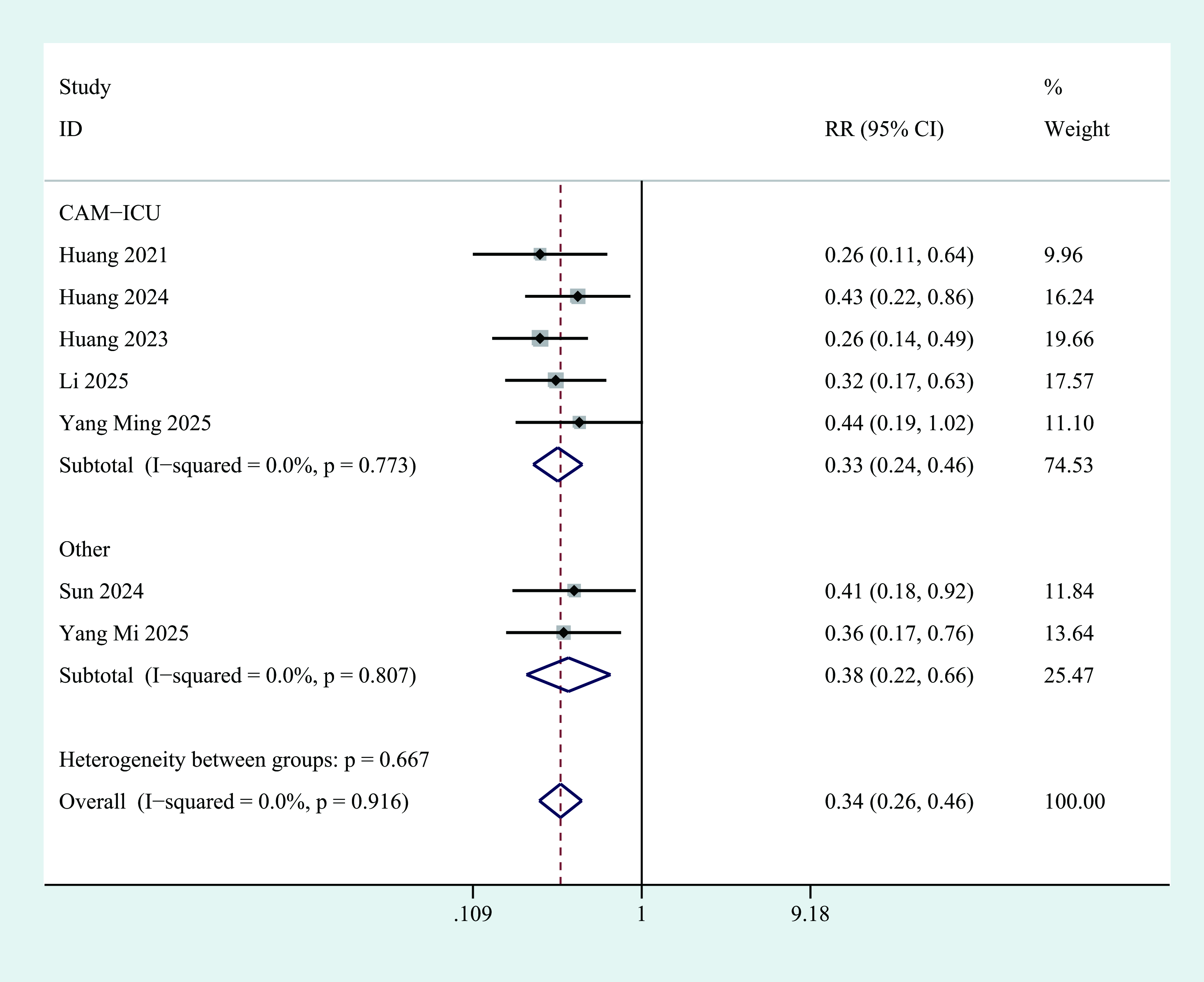

Supplement: Supplementary Figure 6 — Forest plot illustrating the subgroup analysis of incidence of postoperative delirium based on delirium scale. [file Image_6.tif]

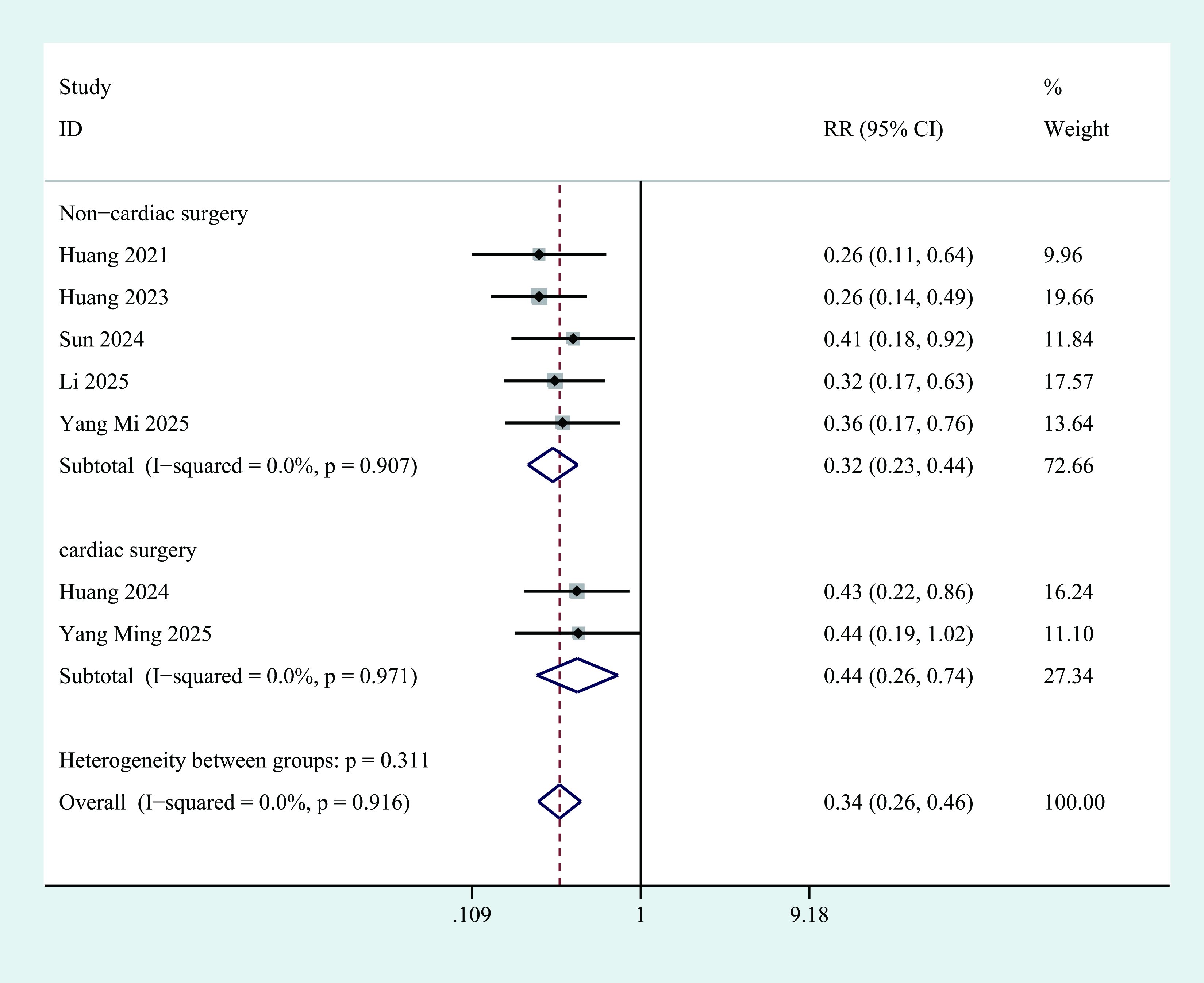

Supplement: Supplementary Figure 7 — Forest plot illustrating the subgroup analysis of incidence of postoperative delirium based on type of surgery. [file Image_7.tif]

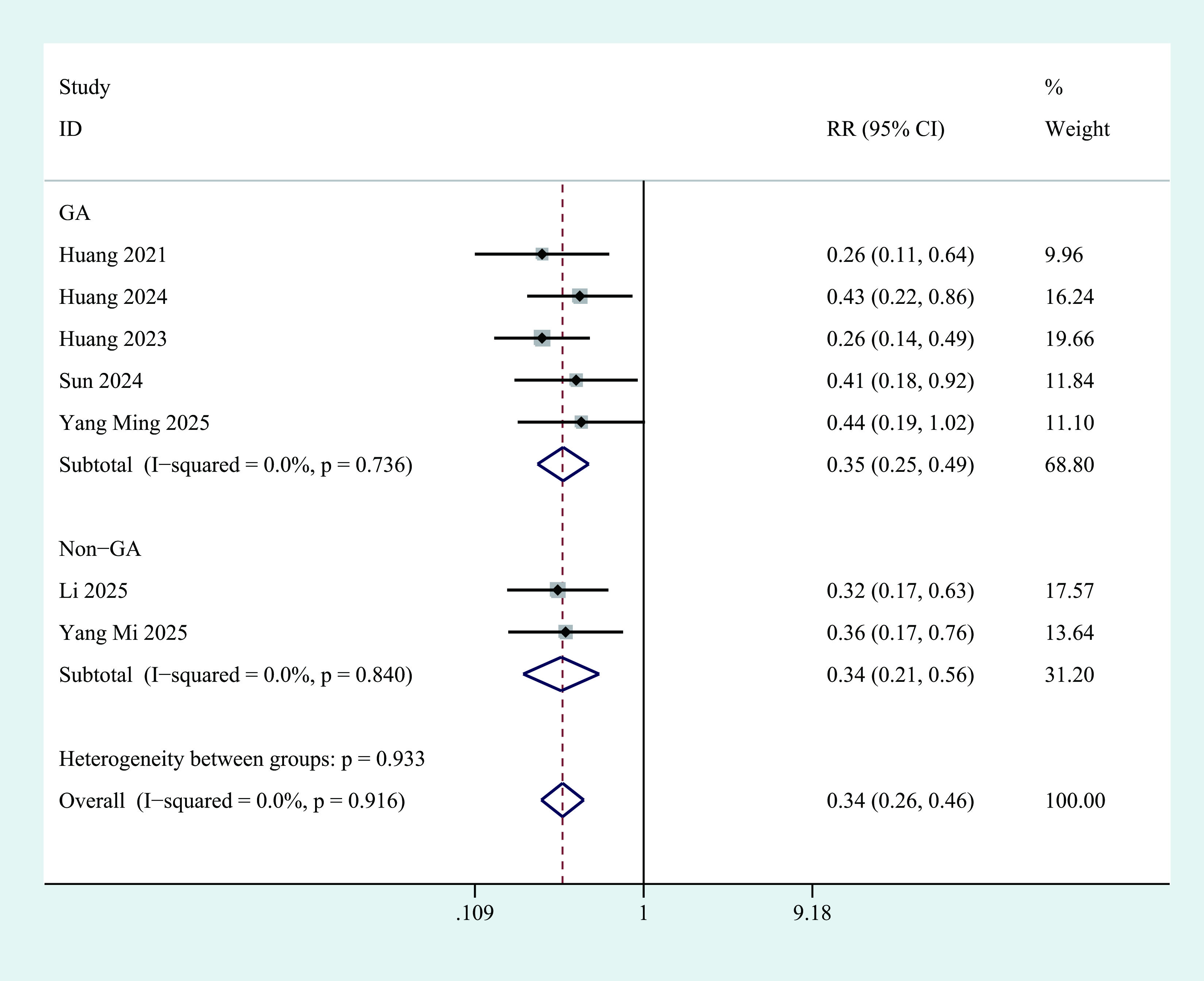

Supplement: Supplementary Figure 8 — Forest plot illustrating the subgroup analysis of incidence of postoperative delirium based on method of anesthesia. [file Image_8.tif]

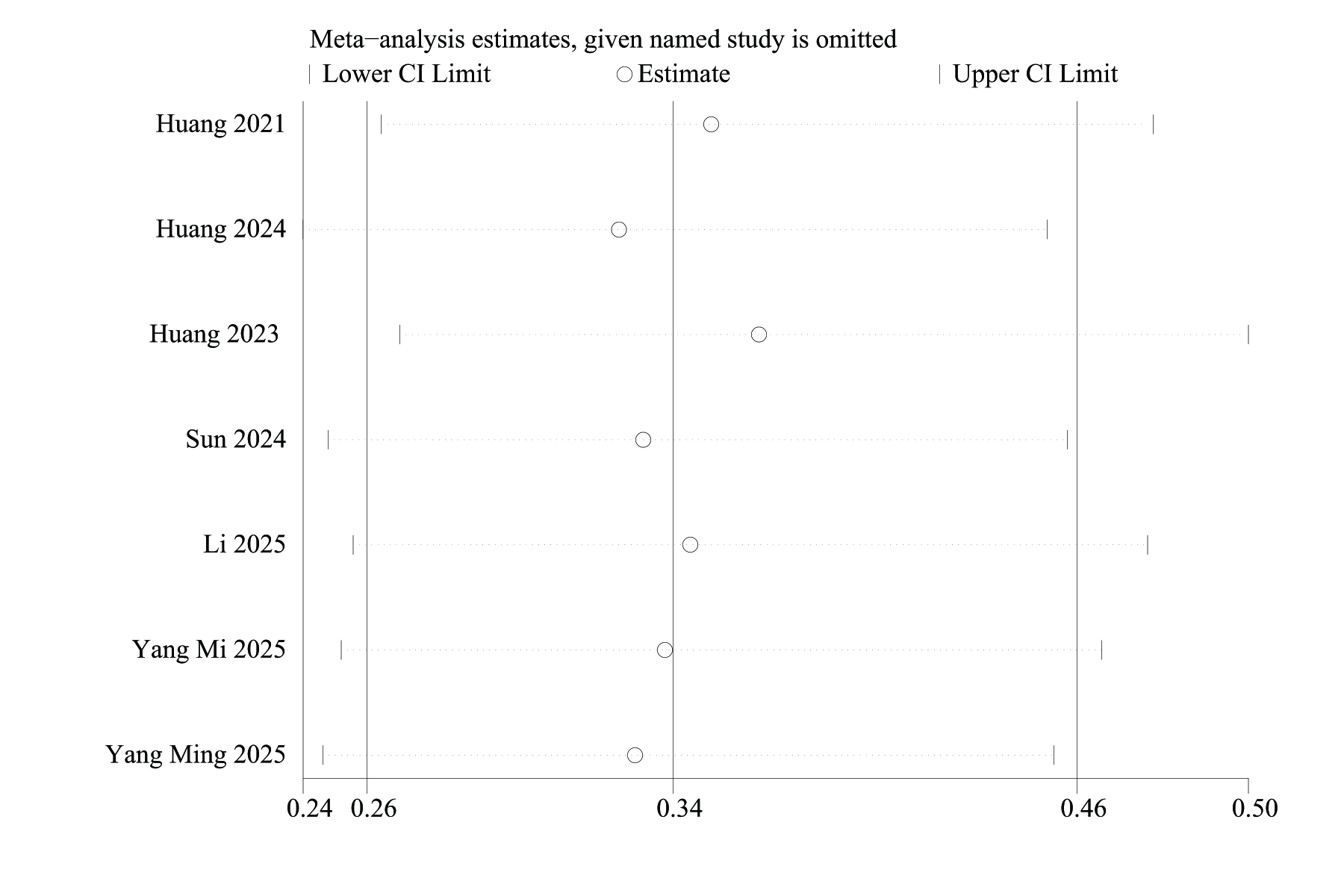

Supplement: Supplementary Figure 9 — Sensitivity analyses of the incidence of postoperative delirium within 3 days of surgery in control group and Insulin group. [file Image_9.tif]
