# Supplementary material for: Intranasal insulin and postoperative delirium in adult surgical patients: a meta-analysis and systematic review of randomized controlled trials
Source: Front Med (Lausanne). 2025 Nov 12;12:1670982. doi: 10.3389/fmed.2025.1670982 (PMC12647034; doi:10.3389/fmed.2025.1670982)
Supplement: Supplementary File 1 — Search strategies for multiple databases. [file Table_1.docx]

**Search strategy in pubmed**

#1 "Delirium"[MeSH Terms] OR ("Delirium"[MeSH Terms] OR "Delirium"[All Fields] OR ("Delirium"[All Fields] AND "mixed"[All Fields] AND "origin"[All Fields]) OR "delirium of mixed origin"[All Fields]) OR ("Delirium"[MeSH Terms] OR "Delirium"[All Fields] OR ("mixed"[All Fields] AND "origin"[All Fields] AND "Delirium"[All Fields])) OR ("Delirium"[MeSH Terms] OR "Delirium"[All Fields] OR ("mixed"[All Fields] AND "origin"[All Fields] AND "deliriums"[All Fields])) OR ("Delirium"[MeSH Terms] OR "Delirium"[All Fields] OR ("subacute"[All Fields] AND "Delirium"[All Fields]) OR "subacute delirium"[All Fields]) OR ("Delirium"[MeSH Terms] OR "Delirium"[All Fields] OR ("deliriums"[All Fields] AND "subacute"[All Fields])) OR ("Delirium"[MeSH Terms] OR "Delirium"[All Fields] OR ("Delirium"[All Fields] AND "subacute"[All Fields])) OR ("Delirium"[MeSH Terms] OR "Delirium"[All Fields] OR ("subacute"[All Fields] AND "deliriums"[All Fields]))

#2 "Insulin"[MeSH Terms] OR ("Insulin"[MeSH Terms] OR "Insulin"[All Fields] OR ("Insulin"[All Fields] AND "regular"[All Fields]) OR "insulin regular"[All Fields]) OR ("Insulin"[MeSH Terms] OR "Insulin"[All Fields] OR ("regular"[All Fields] AND "Insulin"[All Fields]) OR "regular insulin"[All Fields]) OR ("Insulin"[MeSH Terms] OR "Insulin"[All Fields] OR ("soluble"[All Fields] AND "Insulin"[All Fields]) OR "soluble insulin"[All Fields]) OR ("Insulin"[MeSH Terms] OR "Insulin"[All Fields] OR ("Insulin"[All Fields] AND "soluble"[All Fields]) OR "insulin soluble"[All Fields]) OR ("Insulin"[MeSH Terms] OR "Insulin"[All Fields] OR "iletin"[All Fields]) OR ("Insulin"[MeSH Terms] OR "Insulin"[All Fields] OR "insulin a chain"[All Fields]) OR ("insulin regular human"[All Fields] OR "Insulin"[MeSH Terms] OR "Insulin"[All Fields] OR "novolin"[All Fields] OR "insulin, isophane"[MeSH Terms] OR ("Insulin"[All Fields] AND "isophane"[All Fields]) OR "isophane insulin"[All Fields] OR "insulin isophane"[All Fields] OR "isophane insulin, human"[MeSH Terms] OR ("isophane"[All Fields] AND "Insulin"[All Fields] AND "human"[All Fields]) OR "human isophane insulin"[All Fields] OR ("Insulin"[All Fields] AND "human"[All Fields] AND "isophane"[All Fields]) OR "insulin human isophane"[All Fields] OR "nph insulin human"[All Fields]) OR ("Insulin"[MeSH Terms] OR "Insulin"[All Fields] OR ("sodium"[All Fields] AND "Insulin"[All Fields]) OR "sodium insulin"[All Fields]) OR ("Insulin"[MeSH Terms] OR "Insulin"[All Fields] OR ("Insulin"[All Fields] AND "sodium"[All Fields]) OR "insulin sodium"[All Fields]) OR ("Insulin"[MeSH Terms] OR "Insulin"[All Fields] OR "insulin b chain"[All Fields]) OR ("Insulin"[MeSH Terms] OR "Insulin"[All Fields] OR "chain insulin b"[All Fields])

#3 #1 AND #2

**Web of Science**

#1 TS=(delirium OR "delirium of mixed origin" OR "subacute delirium")

#2 TS=(insulin OR "insulin regular" OR "regular insulin" OR "soluble insulin" OR "insulin soluble" OR iletin OR "insulin a chain" OR novolin OR "isophane insulin" OR "insulin isophane" OR "human isophane insulin" OR "nph insulin" OR "sodium insulin" OR "insulin b chain")

#3 #1 AND #2

**Cochrane Library (CENTRAL)**

#1 MeSH descriptor: [Delirium] this term only

#2 (delirium):ti,ab,kw OR "delirium of mixed origin":ti,ab,kw OR "subacute delirium":ti,ab,kw

#3 #1 OR #2

#4 MeSH descriptor: [Insulin] this term only

#5 (insulin):ti,ab,kw OR "insulin regular":ti,ab,kw OR "regular insulin":ti,ab,kw OR "soluble insulin":ti,ab,kw OR "insulin soluble":ti,ab,kw OR (iletin):ti,ab,kw OR "insulin a chain":ti,ab,kw OR (novolin):ti,ab,kw OR "isophane insulin":ti,ab,kw OR "nph insulin":ti,ab,kw OR "sodium insulin":ti,ab,kw OR "insulin b chain":ti,ab,kw

#6 #4 OR #5

#7 #3 AND #6

**Embase**

#1 exp delirium/

#2 delirium.ti,ab,kw OR "delirium of mixed origin".ti,ab,kw OR "subacute delirium".ti,ab,kw

#3 #1 OR #2

#4 exp insulin/

#5 insulin.ti,ab,kw OR "insulin regular".ti,ab,kw OR "regular insulin".ti,ab,kw OR "soluble insulin".ti,ab,kw OR "insulin soluble".ti,ab,kw OR iletin.ti,ab,kw OR "insulin a chain".ti,ab,kw OR novolin.ti,ab,kw OR "isophane insulin".ti,ab,kw OR "insulin isophane".ti,ab,kw OR "human isophane insulin".ti,ab,kw OR "nph insulin".ti,ab,kw OR "sodium insulin".ti,ab,kw OR "insulin b chain".ti,ab,kw

#6 #4 OR #5

#7 #3 AND #6

**WHO-approved trial registry (WHO ICTRP)**

#1 Disease/condition: "Delirium" OR "Delirium of Mixed Origin" OR "Subacute Delirium" OR "Postoperative Delirium" OR "ICU Delirium"

#2 Intervention: "Intranasal Insulin" OR "Nasal Insulin" OR "Insulin" OR "Insulin Regular" OR "Soluble Insulin" OR "Isophane Insulin" OR "Insulin Sodium"

#3 #1 AND #2

**OpenGrey**

#1 "Delirium" OR "Delirium of Mixed Origin" OR "Subacute Delirium" OR "Postoperative Delirium" OR "ICU Delirium" [Title/Abstract]

#2 "Intranasal Insulin" OR "Nasal Insulin" OR "Insulin" OR "Insulin Regular" OR "Soluble Insulin" OR "Isophane Insulin" OR "Insulin Sodium" OR "Insulin A Chain" OR "Insulin B Chain" [Title/Abstract]

#3 #1 AND #2
