# Supplementary material for: Intranasal insulin and postoperative delirium in adult surgical patients: a meta-analysis and systematic review of randomized controlled trials
Source: Front Med (Lausanne). 2025 Nov 12;12:1670982. doi: 10.3389/fmed.2025.1670982 (PMC12647034; doi:10.3389/fmed.2025.1670982)
Supplement: Supplementary File 2 — Supplementary Tables 1, 2. [file Table_2.docx]

**Supplement Table 1. Grading of Recommendations Assessment, Development, and Evaluation (GRADE) assessment of the level of certainty of the evidence.**

|  | **Number of patients** | | **Effect** | | **Certainty of the evidence (GRADE)** | **Comments** |
| --- | --- | --- | --- | --- | --- | --- |
|  | **Insulin group** | **Saline group** | **Relative or WMD**  **(95%CI)** | **Absolute** |  |  |
| **Postoperative delirium (7 RCTs)** | 55/417  (13.2%) | 127/347  (36.6%) | RR 0.35 (0.26 to 0.46) | 238 fewer per 1000 (from 271 fewer to 198 fewer) | ⊕⊕⊕O MODERATE | a |
| **MMSE (2 RCTs)** | 99 | 100 | WMD 0.99 (0.51 to 1.47) | WMD 0.99 higher (0.51 higher to 1.47 higher) | ⊕⊕⊕O MODERATE | a |
| **Preoperative glucose level (5 RCTs）** | 226 | 226 | WMD -0.00 (-0.16 to 0.16) | WMD 0.00 lower (0.16 lower to 0.16 higher) | ⊕⊕⊕O MODERATE | a |
| **Postoperative glucose level (5 RCTs）** | 226 | 226 | WMD -0.02 (-0.27 to 0.24) | WMD 0.02 lower (0.27 lower to 0.24 higher) | ⊕⊕⊕O MODERATE | a |
| **Pain scores on postoperative day 1 (5 RCTs)** | 222 | 221 | WMD -0.36 (-0.77 to 0.05) | WMD 0.36 lower (0.77 lower to 0.05 higher) | ⊕⊕OO LOW | a, b |
| **Pain scores on postoperative day 2 (5 RCTs)** | 182 | 181 | WMD -0.00 (-0.19 to 0.19) | WMD 0.00 lower (0.19 lower to 0.19 higher) | ⊕⊕⊕O MODERATE | a |
| **Pain scores on postoperative day 3 (5 RCTs)** | 222 | 221 | WMD 0.42 (-0.43 to 1.27) | WMD 0.42 higher (0.43 lower to 1.27 higher) | ⊕⊕OO LOW | a, b |

**Explanations**

a. The proportion of information from trials with concerns about the risk of bias may affect the interpretation of the results.

b. High heterogeneity (I2 > 50%). Downgraded by one level for inconsistency

**GRADE Working Group grades of evidence:
High certainty**: We are very confident that the true effect lies close to that of the estimate of the effect
**Moderate certainty**: We are moderately confident in the effect estimate: The true effect is likely to be close to the estimate of the effect, but there is a possibility that it is substantially different
**Low certainty**: Our confidence in the effect estimate is limited: The true effect may be substantially different from the estimate of the effect
**Very low certainty**: We have very little confidence in the effect estimate: The true effect is likely to be substantially different from the estimate of effect

**Supplement Table 2: Sensitivity analyses of postoperative delirium**

| **Category** | **Number of Trails** | **Risk Ratio (RR) and 95% Confidence Interval (CI)** | **P value**  **(RR)** | **I-squared (relative heterogeneity, %)** | **Test for subgroup differences**  **P value** |
| --- | --- | --- | --- | --- | --- |
| **Postoperative Care** |  |  |  |  | 0.311 |
| Postoperative transfer to the ICU | 2 | 0.44 (0.26, 0.74) | <0.01 | 0 |  |
| Postoperative transfer to the general ward | 5 | 0.32 (0.23, 0.44) | <0.01 | 0 |  |
| Overall combined result | 7 | 0.34 (0.26, 0.46) | <0.01 | 0 |  |
|  |  |  |  |  |  |
| **Research Team** |  |  |  |  | 0.544 |
| Studies by the same research team | 3 | 0.31 (0.21, 0.47) | <0.01 | 0 |  |
| Studies by different research teams | 4 | 0.37 (0.26, 0.54) | <0.01 | 0 |  |
| Overall combined result | 7 | 0.34 (0.26, 0.46) | <0.01 | 0 |  |
|  |  |  |  |  |  |
| **Anesthesia Techniques** |  |  |  |  | 0.559 |
| Non-GA | 2 | 0.34 (0.21, 0.56) | <0.01 | 0 |  |
| GA | 3 | 0.30 (0.19, 0.46) | <0.01 | 0 |  |
| GA with CPB | 2 | 0.44 (0.26, 0.74) | <0.01 | 0 |  |
| Overall combined result | 7 | 0.34 (0.26, 0.46) | <0.01 | 0 |  |
|  |  |  |  |  |  |
| **Diabetes status** |  |  |  |  | 0.825 |
| Studies enrolled patients with diabetes | 3 | 0.36 (0.23, 0.55) | <0.01 | 0 |  |
| Studies excluded patients with diabetes | 4 | 0.34 (0.23, 0.48) | <0.01 | 0 |  |
|  |  |  |  |  |  |

ICU:Intensive Care Unit; GA: General Anesthesia; non-GA: Non-General Anesthesia; GA with CPB: General Anesthesia with Cardiopulmonary Bypass; A P-value less than 0.05 indicates a statistically significant difference.
